# Enabling Biomolecular Simulations with Neural Network Potentials in GROMACS

**DOI:** 10.1021/acs.jctc.6c00808

**Published:** 2026-08-20

**Authors:** Lukas Müllender, Berk Hess, Erik Lindahl

**Affiliations:** † Science for Life Laboratory and Swedish e-Science Research Center, Department of Applied Physics, 7655 KTH Royal Institute of Technology, 171 65 Solna, Sweden; ‡ Science for Life Laboratory, Department of Biochemistry and Biophysics, 7675 Stockholm University, 171 65 Solna, Sweden; § Department of Physics, Chemistry and Biology, Linköping University, 581 83 Linköping, Sweden; ∥ Department Chemistry, University of Illinois at Urbana−Champaign, Urbana, Illinois 61801, United States

## Abstract

Neural network potentials (NNPs) are rapidly changing the landscape of state-of-the-art molecular dynamics (MD) simulations. To make full use of this development, the community needs flexible, easy-to-use interfaces firmly integrated with existing methodologies. To address this, we present an interface for hybrid machine learning/molecular mechanics (ML/MM) simulations implemented in the widely used MD code GROMACS. The interface enables NNPs trained in the PyTorch framework to contribute energies and forces during MD simulations, either for selected subsets or entire molecular systems. By defining a flexible set of model inputs and outputs, the interface is agnostic to specific NNP architectures and can accommodate a wide range of descriptor-based and message-passing models. In particular, the design integrates NNP inference seamlessly into the extensive GROMACS molecular simulation ecosystem, providing users with the capability to straightforwardly combine NNPs with existing advanced sampling and free energy workflows. We demonstrate the capabilities of the interface using several representative applications, including enhanced sampling of peptide torsional free energy landscapes, absolute solvation free energy calculations, and protein–ligand simulations. We also run performance benchmarks on water boxes for several different NNP architectures. Our interface is available in recent GROMACS releases, and we believe it will provide a practical foundation for incorporating machine learning potentials into production MD simulations of biomolecular systems.

## Graphical abstract

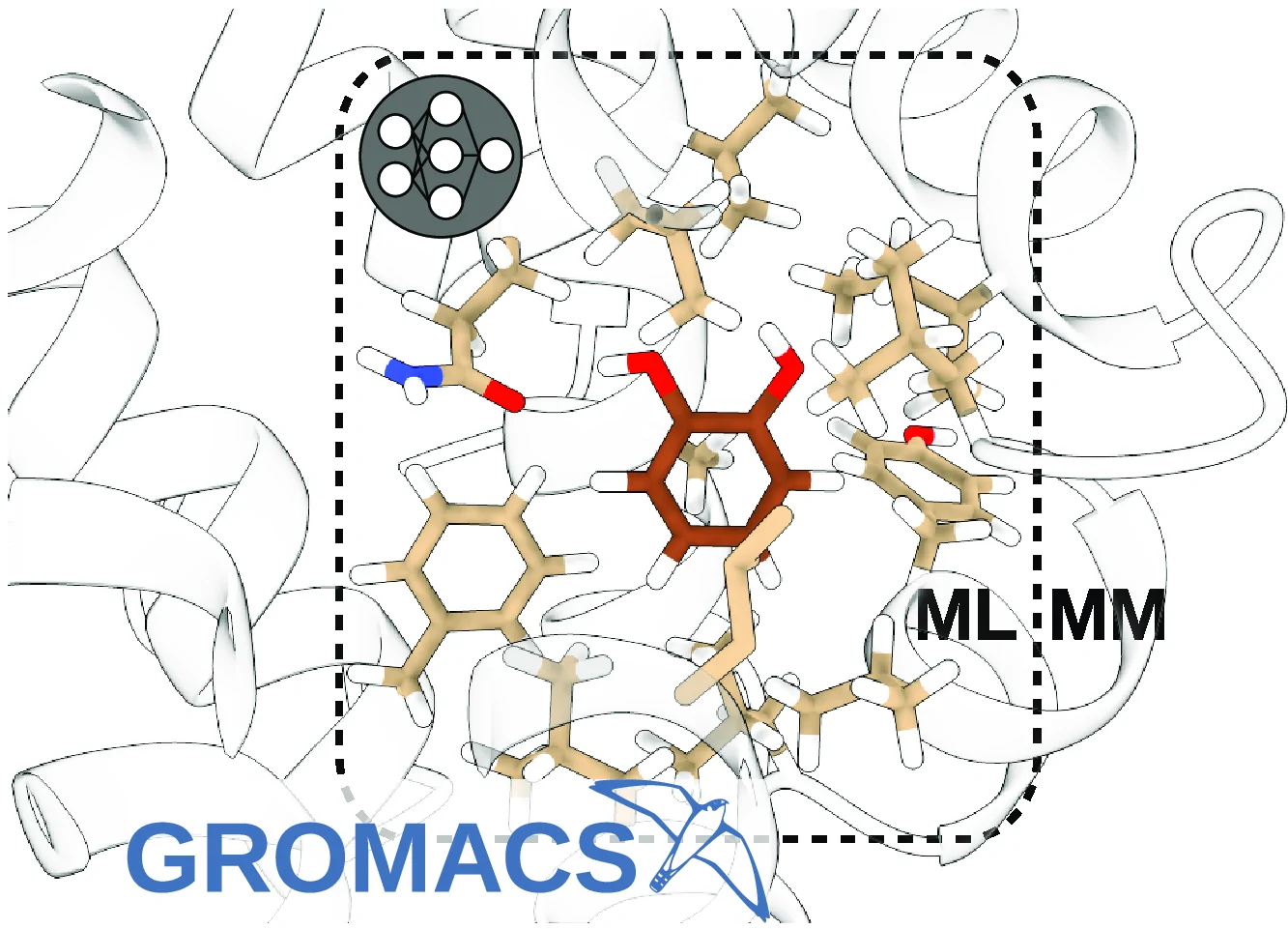

## Introduction

Since their development in the 1970s,^1^ hybrid quantum mechanical/molecular mechanical (QM/MM) simulations have established themselves as a powerful computational tool, combining the enthalpic accuracy of electronic structure methods with the powerful sampling capabilities of molecular dynamics (MD) simulations.^2^ They provide an ideal trade-off between accuracy and efficiency especially in biomolecular systems, which are highly heterogeneous and often necessitate large solvent boxes best treated classically.^3^ However, QM/MM simulations remain computationally intensive as a result of the bottleneck of *ab initio* QM calculations, even for QM regions of moderate size, making the treatment of many biological and chemical phenomena costly and time-consuming.

In recent years, the development of a class of machine learning (ML) models called machine learning interactomic potentials (MLIPs), or more specifically neural network potentials (NNPs), is promising to address this issue, by reproducing energies and forces with QM accuracy at a significantly reduced computational cost.^4,5^ Since the seminal work of Behler and Parrinello,^6^ many different architectures have demonstrated excellent predictive accuracy on a wide range of available benchmarks, including ANI,^7,8^ AIMNet,^9,10^ MACE,^11^ PhysNet,^12^ NequIP^13^ and others.

Generally, applications of MD simulations with NNPs fall into two main classes, each with their own challenges: Full ML simulations, where the entire system is modeled by an NNP similar in spirit to *ab initio* MD, and hybrid ML/MM simulations akin to QM/MM approaches. The first has found use in biomolecular applications^14–16^ and particularly in materials science, where they are now the defacto standard due to the lack of broadly applicable empirical force fields.^17^ However, these simulations face challenges related to high compute and memory requirements, which requires scaling to multiple GPUs,^18,19^ as well as the accurate incorporation of long-range interactions, which is an area of ongoing research.^20–23^

In the context of biomolecular simulations, where widely applicable force fields and water models are available, employing a more expensive model for the description of e.g., solvent–solvent interactions which are ultimately of lesser interest might not be the most efficient use of computational resources. For this reason, it remains desirable to perform hybrid ML/MM simulations, where only a central region of interest is modeled with the NNP, while the rest of the system is treated with classical force fields. Such approaches have already shown promise for example in its application to conformational and binding free energies of protein–ligand complexes,^24,25^ alchemical free energy calculations^25–27^ and vibrational spectroscopy.^28–30^ These works give rise to the nontrivial question of how to treat long-range atomic interactions across the ML–MM boundary. Many works have implemented a scheme analogous to mechanical embedding (ME),^24,25,31,32^ which is straightforward to implement with NNPs but known to perform insufficiently in many QM/MM applications.^2,3^ There, the state-of-the-art is represented by electrostatic embedding, which has been adapted to NNPs by the EMLE method^33,34^ and others.^28,29,35^

When it comes to the practical application of this breadth of different competing approaches in high-performance MD simulation engines, there is a clear need for flexible software interfaces that can accommodate the architectural diversity, while keeping compatibility with existing workflows. While direct implementations and targeted optimizations exist for specific architectures,^15,32^ a more viable alternative is to rely on popular ML frameworks like PyTorch^36^ or JAX.^37^ This strategy has been taken by several implementations of NNP interfaces in popular MD codes like ASE,^38^ OpenMM,^31^ LAMMPS^39^ and Amber.^40^ Furthermore, packages that offer training capabilities for specific NNP architectures often also implement interfaces to MD engines of their choice, like MACE,^11^ DeePMD-kit^41–43^ and chemtrain-deploy.^19^

In this paper, we present a general and flexible interface for performing ML/MM simulations in the widely used molecular simulation toolkit GROMACS.^44^ Our interface, which we call nnpot, allows for NNPs trained within the popular PyTorch framework to contribute to the force calculation for an arbitrary subset of atoms in the system, or its entirety. It is actively maintained as part of the main GROMACS repository since its 2025 release, with the 2026 version adding significant updates to model compatibility and applicability. To be used with the interface, an arbitrary NNP only has to conform to a user-defined set of expected inputs and outputs, allowing for a high degree of flexibility. While this approach allows for straightforward customization, it might provide a somewhat high barrier of entry for users less familiar with machine learning frameworks, which is why we also provide a GitHub repository with an extensive how-to guide and several examples. We note that while our examples focus on popular NNP architectures like ANI and MACE, our interface is designed to be general and to work with any NNP that conforms to the API set by the interface.

After briefly reviewing the main concepts of NNPs and ML/MM simulations, as well as an overview of the implementation and main features of our interface, we showcase its capabilities on a range of different applications: To check the compatibility with enhanced sampling techniques available in GROMACS, we compute the torsional free energy profile of alanine dipeptide in water; next, we perform absolute alchemical free energy calculations to determine the solvation free energy of a selected subset of molecules from the FreeSolv database;^45^ to study the effects of the size of the ML region on protein–ligand binding, we consider the example case of catechol bound to the lysozyme L99A/M102Q mutant; last, we compare the performance scaling of a range of popular state-of-the-art NNPs.

## Theory

### Neural Network Potentials in ML/MM Simulations

In an ML/MM simulation, the total energy of the system can be expressed analogous to a QM/MM simulation as

1 $$E_{\mathrm{tot}}=E_{\mathrm{ML}}+E_{\mathrm{MM}}+E_{\mathrm{ML-MM}}$$

where *E*_ML_ describes the energy of the ML subsystem predicted by the NNP, *E*_MM_ the energy of the remainder of the system described at the MM level, and *E*_ML–MM_ describes a coupling term to encompass all interactions between the ML and MM regions.

The energy *E*_ML_, as predicted by the NNP, is a function of the atomic coordinates {***r***_*i*_^ML^} and nuclear charges {*Z*_*i*_^ML^}. To propagate the simulation, forces acting on the ML atoms also need to be inferred. While approaches have been proposed that predict forces directly,^46^ a much more common approach is to derive them from the energy prediction to guarantee energy conservation by construction

2 $$F_{i}^{\mathrm{ML}}=-\nabla_{i}E_{\mathrm{ML}}$$

for a force acting on atom *i* of the ML region.

For the description of the coupling term *E*_ML–MM_, multiple possibilities exist, inspired by existing schemes from QM/MM approaches.^3^ The simplest of these is termed mechanical embedding, as it practically leaves all pairwise interactions between atoms in the ML and MM regions on the MM level

3 $$E_{\mathrm{ML-MM}}=\sum_{i\in ML}\sum_{j\in MM}E_{\mathrm{ij}}^{Coul}+E_{\mathrm{ij}}^{LJ}$$

where *E*_*ij*_^Coul^ and *E*_*ij*_^LJ^ are the Coulomb and Lennard–Jones interactions between particles *i* and *j*, determined from fixed partial charges and Lennard-Jones parameters taken from the MM force field.

A more sophisticated strategy, and most widely used in QM/MM simulations, is the electrostatic embedding scheme, which can account for polarization effects on the ML region as a result of the surrounding MM atoms.^1^ While in QM/MM, this is done by including the MM atoms as point charges in the QM Hamiltonian, the implementation of such a scheme for NNPs is less straightforward. A number of approaches proposed in the literature not only predict energies and forces on ML atoms, but also their *in vacuo* partial charges, where the influence of the MM atoms is modeled by adding static and induced polarization corrections.^29,33,47^ However, other schemes exist based on the explicit inclusion of the electrostatic field generated by the MM atoms, or the addition of a polarizable buffer region.^35,48,49^

Special attention needs to be given toward boundaries between ML and MM regions that cut through covalent bonds. In QM/MM simulations, a common strategy is to introduce a so-called link atom at an appropriate position along the QM–MM bond, which is only present in the QM subsystem and serves to complete the valence on the adjacent QM atom.^50,51^ A similar strategy can be employed in ML/MM simulations, where broken covalent bonds may lead to local chemical environments far outside the training regime of the NNP model. However, it should be noted that such a scheme is not without flaws and has to be applied with care, even in the case of QM/MM.^52^

## Methods

### The nnpot Interface in GROMACS

The nnpot interface for performing ML/MM simulations in GROMACS is based on the modular MDModules framework, which greatly simplifies the calculation and integration of external forces on parts of the simulation system. The general workflow of such a simulation is illustrated in Figure 1. It can be simply invoked by adding a new section nnpot-active = yes to the.mdp file during system preparation (see Figure 1). In its current form, it allows the user to specify a path to a NNP model that has been trained in PyTorch and exported via its TorchScript functionality. This enables us to take advantage of PyTorch’s C++ API LibTorch and load models directly in GROMACS at runtime, without the added overhead of the Python interpreter. A similar approach is taken also by TorchANI-Amber^40^ and OpenMM/TorchForce.^31^

**Figure 1:**
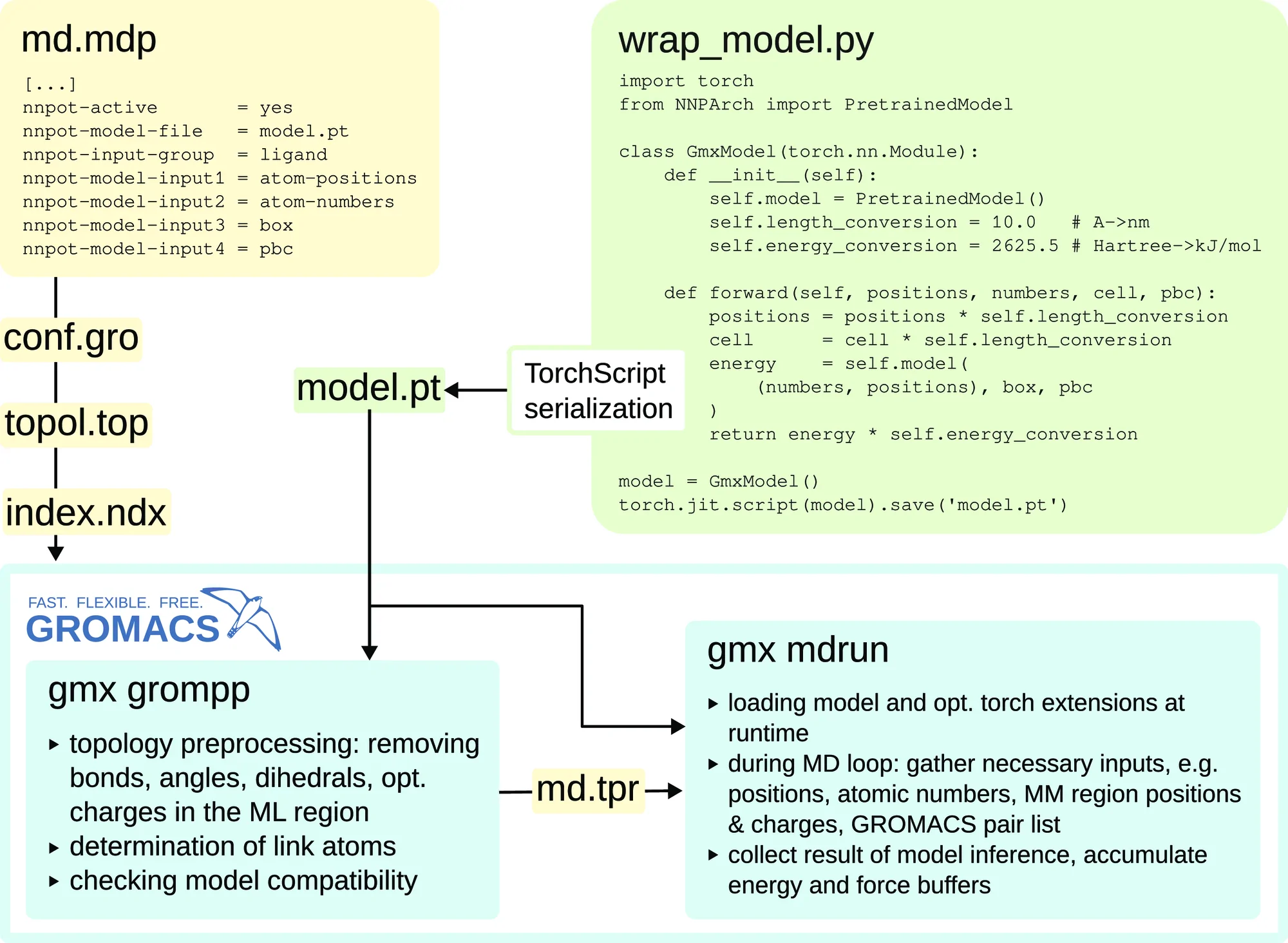
Schematic workflow of an MD simulation using the nnpot interface in GROMACS. The schematic shows the boilerplate code needed to wrap an example model PretrainedModel from an example NNPArch package for use with the interface. The model is exported via TorchScript and loaded within the interface at simulation runtime. Only minimal adjustments to existing simulation input files are needed.

Furthermore, multiple inputs to the model can be specified, such as atomic numbers, positions, the simulation box and type of periodic boundary conditions. The 2026 version adds the internal pair-list built by GROMACS, as well as the positions and charges of MM atoms as available inputs. The ML subsystem on which to apply the NNP is easily specified using an index group, which could be one of the default groups (such as system or non-water) or a custom index group supplied by a.ndx file. If such a selection leads to cut covalent bonds between ML and MM regions, virtual link atoms are introduced automatically, at a fixed distance from the ML atom along the bond to the ML atom, according to

4 $$r_{L}=r_{\mathrm{ML}}+d_{\mathrm{ML}-L}\frac{r_{\mathrm{MM}}-r_{\mathrm{ML}}}{\left|r_{\mathrm{MM}}-r_{\mathrm{ML}}\right|}$$

with link, ML and MM atom positions **r**_L_, **r**_ML_ and **r**_MM_, respectively. Link atom type and distance *d*_ML–L_ can be specified by the user, defaulting to hydrogen and *d*_ML–L_ = 1 Å, respectively. The link atom is treated as a regular ML atom, and the resulting force on it redistributed according to the chain rule.

By itself, the interface currently only allows for simulations with the mechanical embedding (ME) scheme. However, with the addition of positions and charges on MM atoms as an available input in the 2026 version, it is possible to implement an electrostatic embedding (EE) scheme within the NNP model itself, as is done for example in the EMLE model.^34^ In this case, the model can additionally add a force to the MM atoms, calculated via *in vacuo* charges of the ML region additionally predicted by the NNP and used for evaluating the long-range electrostatic interactions, along with a polarization correction.^29,33^ If in use, the interface will automatically remove all classical partial charges of the ML region.

We also note that the model applied in the ML/MM simulation need not be a NNP in the usual sense, but could be used to apply e.g., a harmonic potential on every atom. In such cases, where the analytic form of the atomic gradients is known, forces can be supplied directly by the model to be used by the interface. For detailed information on the use and capabilities of the interface, we refer the user to the GROMACS reference manual.^53^

### Simulation Details

#### Neural Network Potentials

All MD simulations in this paper were produced using GROMACS 2025.4 ^44^ compiled with LibTorch (varying versions), with the exception of the simulations using MACE and EMLE models using a development version of GROMACS 2026. For the simulations using the ANI2x NNP, the corresponding pretrained model from the TorchANI package (v2.2.4)^54^ was used, in combination with the CUDA-optimized implementation of the NNPOps package (v0.6).^32^ For the performance benchmarks on water boxes, we additionally use the new version TorchANI 2.0 (v2.7.1).^55^ For the simulations with MACE (v0.3.14), we use the small-size pretrained foundation model MACE-OFF23.^15^ Other NNP models used in this work are AIMNet2 ^10^ (v0.1.0) and Nutmeg.^56^ The simulations with EMLE were performed using an ANI2x model wrapped with the electrostatic embedding model provided by the EMLE-engine.^34^ For simplicity, we refer to this as the EMLE model throughout this paper.

#### Enhanced Sampling

Enhanced sampling of the torsional free energy (FE) profile of alanine dipeptide in water was carried out using the Accelerated Weight Histogram method (AWH).^57^ To prepare the system, an alanine dipeptide molecule was parametrized with the General Amber Force Field (GAFF)^58^ and solvated with 544 TIP3P^59^ water molecules. Energy minimization was performed for 10,000 steps, after which the system was allowed to equilibrate for 100 ps in the NVT and 100 ps in the NPT ensemble using stochastic velocity and cell rescaling^60,61^ at 300 K and 1 bar, respectively, as well as a time step of 2 fs. From the equilibrated state, enhanced sampling simulations with AWH were carried out at a time step of 1 fs for 10 ns with a force constant of 8000 kJ mol^–1^ rad^–2^, a diffusion constant of 5 rad^2^/ps and estimated initial error of 10 kJ/mol, biasing the two dihedral angles ϕ and ψ of the peptide. FE profiles were reconstructed using the gmx awh command line tool and filtered with a Gaussian kernel of width 1.5°.

#### Absolute Free Energy Calculations

Free energy calculations for the solvation of small molecules from the FreeSolv database^45^ were carried out using the free energy λ mechanism in GROMACS. The systems were prepared using the topologies provided in the database and solvated with TIP3P water molecules. The systems were then minimized using a steepest descents algorithm for 5000 steps or until converged. Equilibration in the *NPT* ensemble was performed in a single simulation for 100 ps, using a Langevin thermostat with a temperature of 298.15 K and c-rescale pressure coupling at 1.013 bar. All simulations were performed using a time step of 1 fs due to the use of NNPs. Production runs of 2 ns for each λ were carried out in the *NPT* ensemble starting with the fully coupled state, and performing calculations for successive values of λ starting from the last frame of the previous simulation. In this way, simulations for the different lambda values cannot be parallelized, but no separate minimization and equilibration runs at each λ are necessary. Instead, the first 10 ps of each production simulation were discarded from further analysis. Free energy calculations were carried out using the BAR^62^ implementation of the gmx bar command line tool in GROMACS. To avoid issues with overlapping charges as interactions are successively turned off, we decouple Coulomb interactions before LJ interactions, and use a Beutler soft-core LJ potential. The parameters of the thermostat and barostat, as well as the spacing of the λ windows, are chosen to match those used in the reference calculations in the FreeSolv database, to ensure comparability. The procedure as described above was applied uniformly to 30 molecules selected from the FreeSolv database without any per-case adjustments. Among them 20 molecules were selected at random, 5 were selected that had the largest discrepancy between experimental and calculated value reported in the database, and 5 with the smallest discrepancy. This was done to check whether the use of NNPs can improve the calculated SFE values especially in cases where the classical FF fails, without sacrificing accuracy in cases that are well reproduced with classical FFs. Four molecules, where the described procedure led to errors during system preparation, were discarded and replaced with new selections preserving the same criteria. The full selected data set with all experimental and calculated values, as well as selection criteria, is shown in Table S1.

#### Protein–Ligand Binding Simulations

The system of lysozyme L99A/M102Q bound to catechol (PDB entry 1XEP) was prepared using the CHARMM36/CGenFF force field.^63,64^ 10084 TIP3P water molecules and 8 chloride ions were added for solvation and neutralization. System minimization and equilibration was again carried out in an pure MM description as described above, with the addition of position restraints for the protein and ligand atoms during equilibration. From the equilibrated configuration, 4 different simulations were started, one classical MM simulation and three ML/MM simulations, with the ML region comprising: 1. only the ligand in ME; 2. the ligand and the surrounding side chains of protein residues (within 3 Å of the ligand) in ME; 3. only the ligand in EE. For the latter, link atoms were introduced, capping cut covalent bonds between C_α_ and C_β_ atoms with a hydrogen atom at a distance of 1 Å along the bond. Again, we use the NNPOps-optimized version of ANI2x, and EMLE as the electrostatic embedding model. Protein–ligand interaction fingerprints were generated with the ProLIF package.^65^

## Results and Discussion

### Efficient Conformational Enhanced Sampling

The conformational landscape of alanine dipeptide (Figure 2a) has been studied long and widely in the literature as a model system for the torsional changes in the backbone of proteins,^66,67^ and used in the parametrization and validation of classical force fields^68^ and NNP architectures and implementations^29,34,35^ alike. Here, we report the Ramachandran plot of the torsional free energy profile of alanine dipeptide along its dihedral angles ϕ and ψ in Figure 2a. Our results reproduce important secondary structure minima consistently between ML/MM and pure MM simulations, and are in good agreement with those reported previously, both at the ML/MM level and a QM/MM reference at ωB98*x*/6–31G* level of theory.^29,33^

**Figure 2:**
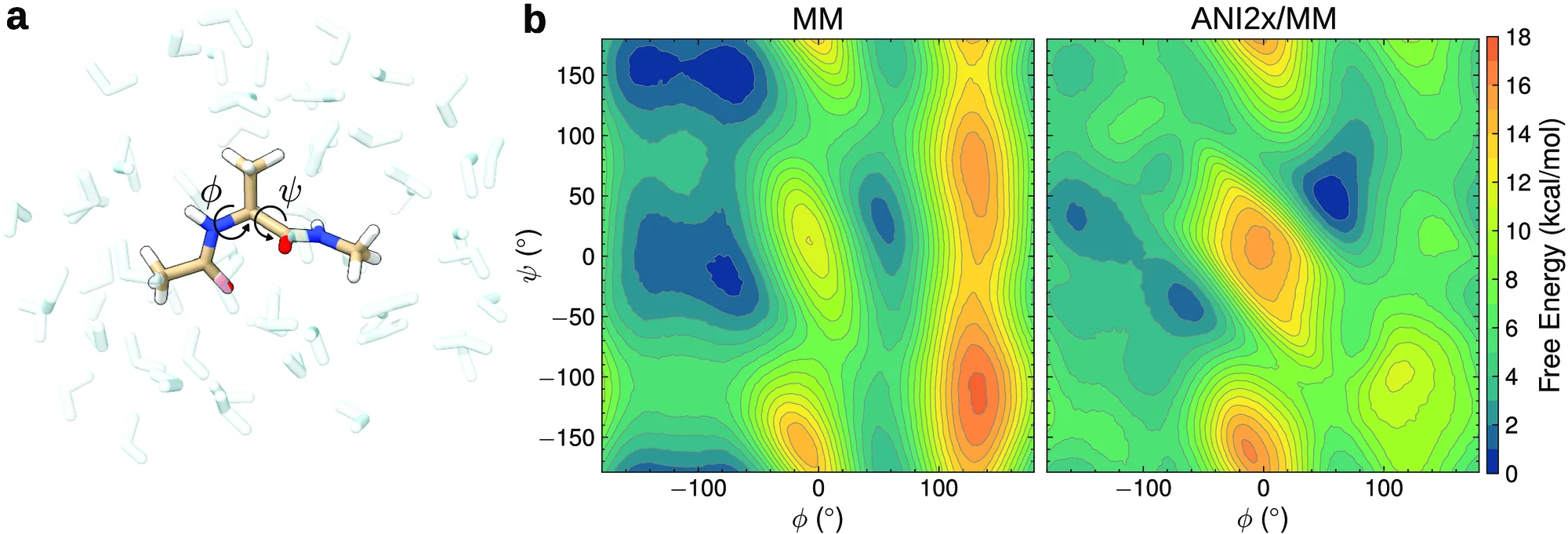
Conformational enhanced sampling with AWH. (a) Structure of an alanine dipeptide molecule solvated in water. (b) Ramachandran plot of the torsional free energy surface of alanine dipeptide in water, calculated using the AWH method for enhanced sampling biasing the two dihedral angles ϕ and ψ. The left side shows the reference calculated at MM level, while the right side shows results with the alanine dipeptide treated with ANI2x.

As a key difference, our results were produced using the AWH method, which has been shown to be highly flexible and effective in calculating free energies in a number of different settings.^57,69^ This demonstrates that our NNP implementation can be readily combined with other sampling methodologies available in GROMACS. Furthermore, because of its efficient implementation, calculating free energies with AWH comes at very little additional cost compared to unbiased ML/MM simulations. In concrete terms, while an MM AWH simulation ran at 941 ns/day, a simulation using ANI2x for the description of the alanine dipeptide ran 42 ns/day, all at a time step of 1 fs. In comparison, an unbiased ML/MM simulation ran at 51 ns/day.

### Solubility of Small Molecules at the ML/MM Level

In addition to conformational enhanced sampling methods, our ML/MM implementation also readily lends itself to alchemical free energy calculations. We showcase this capability by performing SFE calculations on a subset of 30 selected molecules from the FreeSolv database. The computation of SFEs is an important benchmark to gauge our ability to accurately model the binding interactions of proteins and small molecular ligands. Furthermore, they offer a quantitative comparison to experiment, which is critical to assess the quality of our molecular models. In ref 27 the authors perform a systematic study of SFEs on a large subset of the FreeSolv database,^45^ using nonequilibrium switching and the ANI2x NNP to compute end-state corrections to SFE results derived from MM simulations. Here, we instead use an approach more similar to that of ref 45 simulating the entire decoupling process in an ML/MM setup and concentrate on a smaller, 30-molecule subset of the original database.

The SFE results on our 30-molecule subset are illustrated in Figure 3, where we compare the values calculated with the ANI2x NNP for the molecule with the experimental results found in the database and the reference calculations carried out using the GAFF force field parameters using AM1-BCC partial charges. All numerical values for all molecules are reported in the SI, Table S1. In general, the mean absolute error for the ANI results score a mean absolute error (MAE) w.r.t. the experimental results of 1.20 kcal/mol, in contrast to 1.47 kcal/mol for the FreeSolv calculations. In terms of Pearson correlation coefficient, the ANI results score a value of 0.97, the FreeSolv results for the same molecules 0.92. It should be stated, however, that these values present a biased measure of the performance of the two methods, because of the above-mentioned nonstochastic selection methods.

**Figure 3:**
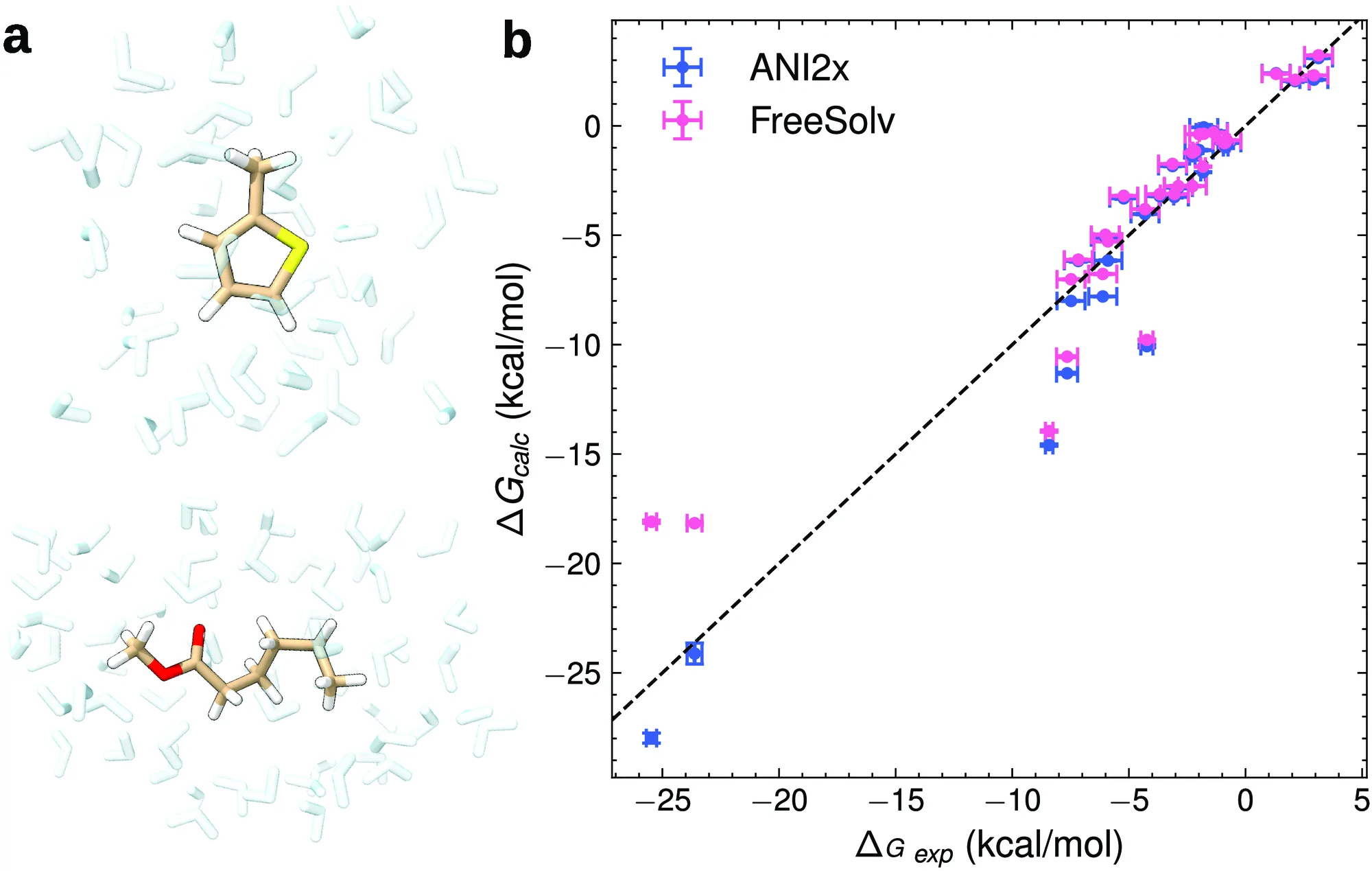
Solubility of small molecules at the ML/MM level. (a) Example molecules from the database, with surrounding water molecules: 2-methylthiophene (top) and methylhexanoate (bottom). (b) Solvation free energies for a subset of small molecules from the FreeSolv database, with inset showing small values of |Δ*G*_solv_|.

Nevertheless, these results suggest that the use of ANI2x can lead to a better estimation of the solvation free energy in some cases compared to GAFF. Crucially, it does not lead to a marked increase in the estimation error for any of the tested molecules. It is important to note that any observed improvement is due solely to an improved description of the intramolecular interactions as the solute–solvent interactions are still treated classically. We also note that in the cases where the reference calculations had the largest errors, the use of the NNP either led to the biggest observed improvements, or none at all. This points to different sources of the prediction error in these cases: where the parametrization of the intramolecular interactions was suboptimal, the NNP is able to significantly increase the prediction quality. In cases where it does not, it can be postulated that insufficiently modeled solute–solvent interactions, i.e., partial charges, are the cause of the error. While the authors in ref 27 report no statistically significant improvements of the overall prediction quality when employing end-state corrections with ANI2x, we cannot definitively corroborate or contradict their findings, as a systematic study is outside of the scope of this work.

We note that in its current form, our NNP interface is only able to perform absolute alchemical free energy calculations. The calculation of relative free energies, where some atoms or entire chemical moieties are transformed into another, would require a more sophisticated single or dual-topology scheme, which is nontrivial to implement in the presence of an NNP. Further improvements in the accuracy of the calculated SFE values could be attained by using a more accurate electrostatic embedding scheme like EMLE, which would require integrating the λ-parameter into the total electrostatic energy calculation of the model. Another possibility would be to describe the system entirely within the NNP, where special care has to be taken that the NNP accurately models long-range electrostatic interactions within itself,^22^ and is able to appropriately describe the annihilation or creation of atoms.^70^

### Effects of ML Region Size and Embedding on Protein–Ligand Binding

In the following, we showcase the applicability of our interface to ML/MM simulations of protein–ligand complexes.^24^ As a test case, we pick the lysozyme L99A/M102Q mutant bound to catechol (see Figure 4a) because of its polar and well-characterized binding site.^71^ We first perform a simulation at the MM level, as well as an ML/MM simulation with the ML region including only the ligand (CAQ). To test the automatic link atom scheme, we then also consider an ML region additionally containing amino acid side chains within 3 Å of the ligand, with the ML/MM boundary bisecting the C_α_–C_β_ bonds of the included residues (CAQ + BS). This setup can also be considered a proxy for the study of enzymatic reactions, where it is often necessary to include residues of the catalytic site in the QM­(ML) region.^72^

**Figure 4:**
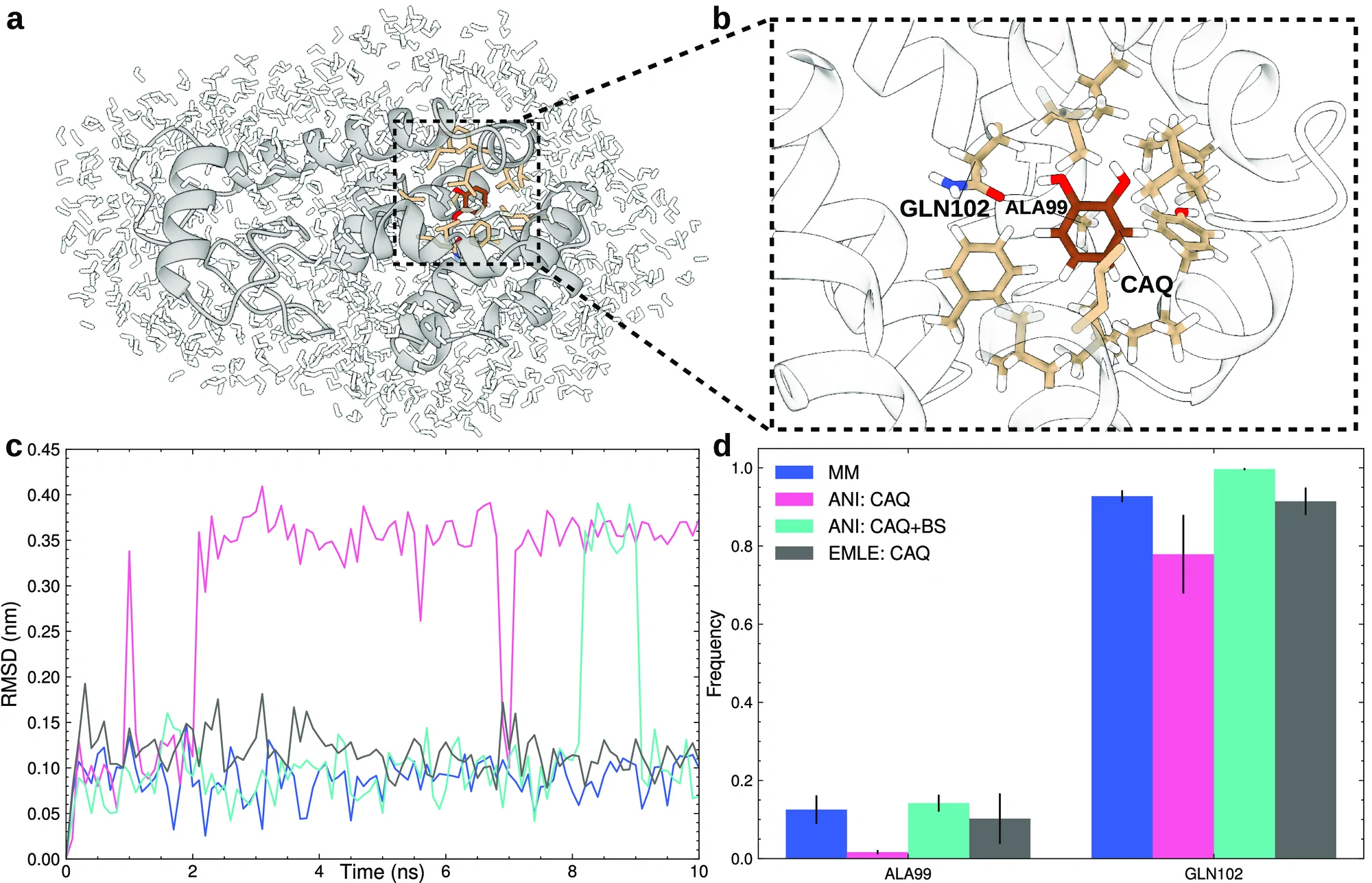
ML region size and embedding scheme affect protein–ligand binding in lysozyme. (a) Structure of lysozyme L99A/M102Q mutant bound to catechol, PDB ID 1XEP, with surrounding water molecules. (b) Close up of the binding site of catechol (CAQ, in sienna), showing the ligand and residues within 3 Å of it (in tan). (c) RMSD calculated with respect to the equilibrated starting structure for one pure MM and three ML/MM simulations: Using ANI2x for the catechol ligand (CAQ), ligand plus binding site (CAQ + BS), and EMLE for catechol. (d) Frequency of hydrogen bond formation between the ligand and two residues of the binding site, ALA99 and GLN102. The figure reports mean values and standard deviations over 3 repeats.

In Figure 4c, we show the root-mean-square deviation (RMSD) of the ligand heavy atoms with respect to the initial configuration, calculated over 10 ns of simulation at the aforementioned levels of description. Results for all three performed replicates are shown in Figure S1. It is apparent that at the MM level, the initial ligand binding pose is stable over the course of the simulation. However, switching to ANI2x for the description of the ligand causes it to preferentially assume a different binding mode. Interestingly, the addition of the surrounding binding side residues into the ML region seems to restabilize the initial pose. This is further confirmed by comparing the frequency of hydrogen bonding interactions between the ligand and protein residues of the binding site, ALA99 and GLN102. We observe a drop in hydrogen bonding frequency for the CAQ simulations, which are restored in the CAQ + BS case. To assess statistical significance of the results, we analyzed results over 3 repeats of the simulations. We note that for the ALA99 residue, the hydrogen bond acceptor is the carbonyl oxygen of the backbone, which is not part of the ML region. As we expect the inclusion of more interactions in the NNP for the CAQ + BS case to be more accurate, this result could be indicative of the limitations of the mechanical embedding scheme used in this application. Empirical FFs are parametrized with bonded and nonbonded parameters to reproduce target observables in conjunction, and simply replacing either of them with a different method is in principle invalid and can be detrimental.

To explore this more closely, we also performed simulations with only the CAQ input to the NNP, but additionally employing the EMLE model^34^ for the ML-MM interactions, which has shown promise in a number of applications.^30,33,34^ Again, we observe hydrogen bonding frequencies of the same magnitude as in the MM case. This could be the effect of stronger interactions between the ligand and binding pocket when accounting for polarization effects on the ML region. A similar effect can be seen when studying solute–solvent effects in an ML/MM simulation of a phenol molecule in water, where previous studies have shown that an electrostatic embedding is necessary to accurately reproduce QM/MM results.^29^ We here additionally confirm that the EMLE model is able to reproduce this result, with the corresponding analysis shown in Figure S2. Taken together, these results suggest that the destabilization of the binding pose could be an artifact of the mechanical embedding scheme.

Thus, when simulating protein–ligand systems, special care should be taken when selecting the molecules and residues to include in the ML region. Due to the excellent scaling of NNP models, increasing the size of the ML region comes at a comparatively modest increase in computational cost. In this case, ML/MM simulations including only the ligand (14 ML atoms) ran at a performance of 58 ns/day, adding the side-chains of residues of the binding site (107 ML atoms, including link atoms) ran at 13 ns/day, and the EMLE model (14 ML atoms) at 9 ns/day, all at a time step of 1 fs. For reference, the MM simulation in GROMACS ran at a performance of 558 ns/day at a time step of 2 fs.

### Performance Scaling of Different NNP Architectures

Lastly, we focus on the computational performance of different NNP architectures when scaling to bulk water systems of different sizes. In contrast to other machine learning applications, a particular requirement for NNPs is low-latency single point inference, to be efficiently used in high-throughput MD simulations and consequently enable longer sampling times. Therefore, it is important for NNP architectures to be designed with both computational and memory efficiency in mind. While scaling NNP inference to multiple GPUs is feasible,^18,19^ we consider it less relevant for hybrid ML/MM simulations, where ML regions are less likely to exceed GPU memory limits, especially with computational hardware rapidly advancing to accommodate the memory requirements of modern large-language models. Thus, we here only focus on single-device inference.

Figure 5 shows the results of our performance scaling experiments. We compare a number of popular state-of-the-art NNPs with available pretrained models: AIMNet2,^10^ ANI2x,^8^ MACE-OFF-small^11,15^ and Nutmeg.^56^ For the ANI2x model, we utilize the recent improved implementation in TorchANI 2.0,^55^ including a CUDA-optimized extension for the calculation of the atomic environments, called cuAEV. Additionally, we consider the NNPOps optimizations on CUDA GPUs for the ANI2x model.^32^ All simulations were run in the NVE ensemble at a time step of 1 fs on an NVIDIA GeForce RTX 3070 GPU. Numerical values for all benchmarks are reported in the SI, Tables S2 and S3. For small systems, we observe that the NNPOps implementation of ANI2x is the fastest model, with close to 90 ns/day (1 ms/step) for the smallest systems. Up to system sizes of 30 atoms, all other models run at a performance of not more than 12 ms/step (8 ns/day at as 1 fs time step). While significantly slower than classical force fields, this performance still marks a speedup of 4 to 5 orders of magnitude compared to QM approaches, where a state-of-the-art approaches for *ab initio* MD simulations of similar size report performances on the order of 100 s/step.^73^ We further observe that the native TorchANI 2.0 implementation of ANI2x is the most computationally efficient model we tested here, being faster than all other models for systems larger than 300 atoms, and the only one that did not run out of device memory for any tested system sizes.

**Figure 5:**
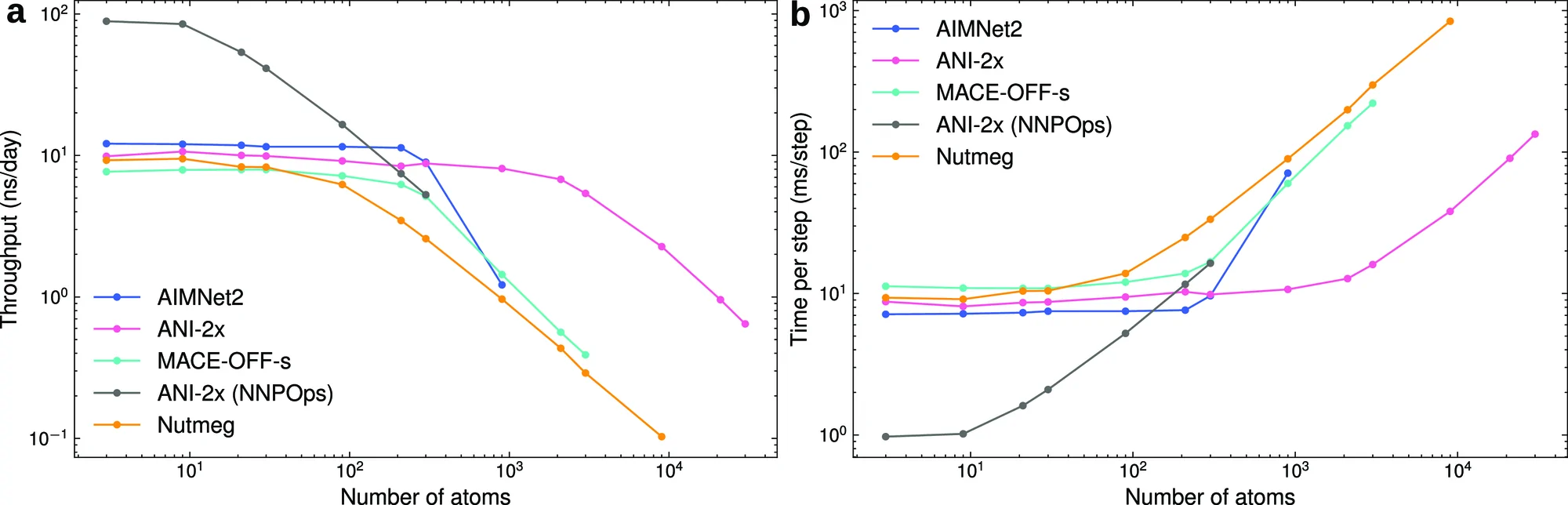
Performance scaling of different NNP architectures on bulk water. Results shown are averages over one 3000 step MD simulation, resetting performance counters after 500 steps. Where no performance data is shown, this indicates that the calculations exceeded GPU memory for these system sizes. The figure shows (a) the throughput in terms of simulated time per wall time in units of ns/day at a 1 fs time step, and (b) the performance in terms of walltime per simulation step in ms. Results were obtained on a NVIDIA RTX 3070 GPU at 32-bit floating point precision.

Note that the performance curves of all models are essentially flat for smaller systems, before exhibiting the expected linear scaling with increasing system size. This plateau is likely due to the combined overhead of repeated CUDA kernel launches and LibTorch API calls, which becomes negligible for larger systems as they transition into a compute-bound regime. In this regime, the performance of a model depends mostly on the amount of launched kernels. This is further supported by the observation that for small NNP region sizes, the NNPOps model is the most performant, almost by a factor of 10. The primary speedup in this implementation is achieved through specialized, fused CUDA operations that efficiently parallelize the 8 neural networks comprising the ensemble ANI2x model while minimizing API interactions. However, the specialized data structures required for this low-latency optimization scale poorly with system size, losing its performance advantage and quickly reaching memory limits. On the other hand, this also indicates that there are potential performance gains to be had in all models by raising (lowering) the small-system plateau, particularly for ML/MM simulation settings. This could be achieved by utilizing modern optimizations like CUDA graphs, and PyTorch’s compile and AOTInductor mechanisms that produce optimized GPU kernels, requiring changes to model architecture.

## Conclusion

In this work, we have introduced a flexible and efficient interface for incorporating neural network potentials into molecular dynamics simulations with GROMACS, called nnpot. By enabling PyTorch-trained models to directly contribute to the force calculations of a subset of atoms or entire systems, this development expands the computational and methodological possibilities for hybrid ML/MM simulations. We have demonstrated that the interface integrates seamlessly with existing GROMACS workflows in a variety of applications, including enhanced sampling techniques like AWH, alchemical free energy methods and protein–ligand simulations. We note that this list is not exhaustive, and that the interface is compatible with most advanced simulation techniques available in GROMACS, such as those implemented the Colvars^74^ and PLUMED^75^ packages.

Our results indicate that neural network potentials in our interface are able to reproduce established reference data and, in some cases, improve upon classical force field descriptions, especially in systems where intramolecular interactions are challenging to capture with traditional parameter sets. At the same time, the presented applications also highlight the limitations of the mechanical embedding scheme and underline the need for further development of electrostatic embedding and free energy schemes for NNPs to extend applicability to a wider range of systems.^34,70,76^

While the computational performance of neural network potentials cannot currently rival that of established and well-validated classical force fields, it is important to highlight their orders-of-magnitude advantage over the *ab initio* QM methods they were trained on. It is also for this reason we believe that in biomolecular contexts, the most promising application of NNPs lies in hybrid ML/MM settings. For these smaller ML system sizes, we observed that nearly all current NNP architectures hit a “speed of light”-like barrier, as computational performance becomes bound by the overhead of LibTorch operation dispatch and kernel launches. Thus, an auspicious avenue for future work lies in the development of direct C++ implementations of specific NNP architectures optimized for inference.

Continued development efforts will also focus on improving the treatment of ML–MM boundaries and long-range electrostatic interactions, improvements regarding computational performance and scaling, and ensuring continued compatibility the rapidly advancing landscape of NNP architectures. Nevertheless, we anticipate that this tool in its current state will accelerate the adoption of ML-based potentials in routine molecular simulations, and create new opportunities for the accurate and efficient modeling of complex biochemical systems at scale.

## Supplementary Material



## Data Availability

Capabilities needed to perform all simulations in this paper are available in the recent 2026 version of GROMACS. Extensive examples and guides on how to export NNPs for use with our interface are available as a GitHub repository at https://github.com/lmuellender/gmx-nnpot-tools, with a persistent record of the version used in this work uploaded to Zenodo at 10.5281/zenodo.19663881.

## Notes

Exact tabulated values for solvation free energy calculations from the FreeSolv database (Section S1, Table S1); RMSD values of catechol bound to lysozyme for all performed replicas (Section S2, Figure S1); solvation structure of a phenol molecule in water, calculated at MM, MACE/MM, ANI2x/MM, and ANI2x-EMLE/MM levels (Section S2, Figure S2); and exact performance numbers of different pretrained NNP models on water boxes of varying sizes (Section S3, Table S2) (PDF)
