# Supplementary material for: Enabling Biomolecular Simulations with Neural Network Potentials in GROMACS
Source: J Chem Theory Comput. 2026 Aug 20;22(17):9134–44. doi: 10.1021/acs.jctc.6c00808 (PMC13564354; doi:10.1021/acs.jctc.6c00808)
Supplement: Supplementary file 1 [file ct6c00808_si_001.pdf]

Supporting Information

Enabling Biomolecular Simulations with Neural Network Potentials in GROMACS

Lukas Müllender,[†] Berk Hess,[†] and Erik Lindahl[*,†,‡,¶,§]

†*Science for Life Laboratory & Swedish e-Science Research Center, Department of Applied Physics, KTH Royal Institute of Technology, Solna 17165, Sweden*

‡*Science for Life Laboratory, Department of Biochemistry and Biophysics, Stockholm University, Solna 17165, Sweden*

¶*Department of Physics, Chemistry and Biology, Linköping University, Linköping 58183, Sweden*

§*Department Chemistry, University of Illinois at Urbana-Champaign, Urbana, IL 61801, United States*

E-mail: erik@kth.se

S1 Solvation Free Energy Calculations on the FreeSolv Database

Table S1: Solvation free energy results for 30 selected molecules from the FreeSolv database. The table reports the molecule ID as given in the database, SFE values ΔG_X and uncertainty σ_X for experimental and predicted values with GAFF (from database) and with ANI2x. The last column indicates whether the respective molecule was selected at random or by largest/smallest deviations between experimental and GAFF-predicted value.

ID	ΔG_{exp}	σ_{exp}	ΔG_{GAFF}	σ_{GAFF}	ΔG_{ANI}	σ_{ANI}	Selection
mobley_1328465	-1.99	0.60	-0.38	0.03	-1.11	0.04	Random
mobley_9705941	-1.38	0.60	-0.30	0.02	-0.26	0.05	Random
mobley_5494918	2.93	0.60	2.31	0.02	2.11	0.05	Random
mobley_3006808	-4.31	0.60	-3.81	0.02	-4.03	0.03	Random
mobley_8723116	-5.90	0.60	-5.28	0.03	-6.16	0.04	Random
mobley_3690931	-2.28	0.60	-2.75	0.03	-2.76	0.02	Random
mobley_1857976	-1.79	0.60	-0.36	0.02	-0.07	0.13	Random
mobley_5538249	-2.16	0.10	-1.15	0.04	-1.21	0.05	Random
mobley_9407874	-2.88	0.60	-2.76	0.03	-2.83	0.05	Random
mobley_3234716	-0.80	0.60	-0.66	0.03	-0.78	0.04	Random
mobley_4218209	-7.48	0.60	-7.02	0.03	-8.00	0.02	Random
mobley_1659169	-7.17	0.60	-6.12	0.03	-6.18	0.05	Random
mobley_9112978	1.31	0.60	2.37	0.02	2.40	0.02	Random
mobley_628951	-2.28	0.12	-1.23	0.04	-1.39	0.08	Random
mobley_2792521	-3.68	0.60	-3.12	0.03	-3.21	0.02	Random
mobley_2371092	-5.22	0.60	-3.20	0.03	-3.32	0.05	Random
mobley_967099	-3.13	0.60	-1.74	0.02	-1.84	0.03	Random
mobley_1873346	-0.90	0.20	-0.79	0.02	-0.79	0.04	Random
mobley_5072416	-6.12	0.60	-6.77	0.03	-7.80	0.02	Random
mobley_4780078	-6.01	0.60	-4.98	0.03	-5.10	0.04	Random
mobley_3047364	-7.65	0.45	-10.55	0.04	-11.31	0.05	Large
mobley_4587267	-23.62	0.32	-18.16	0.08	-24.12	0.48	Large
mobley_1527293	-8.42	0.16	-13.95	0.05	-14.59	0.05	Large
mobley_9571888	-4.23	0.26	-9.78	0.04	-10.07	0.08	Large
mobley_9534740	-25.47	0.22	-18.10	0.07	-27.98	0.23	Large
mobley_6416775	2.13	0.60	2.10	0.03	2.04	0.06	Small
mobley_7869158	-1.82	0.10	-1.86	0.03	-2.12	0.02	Small
mobley_8337977	-3.05	0.60	-3.12	0.03	-3.25	0.04	Small
mobley_3040612	-0.85	0.10	-0.76	0.03	-0.98	0.06	Small
mobley_4375719	3.13	0.60	3.22	0.03	3.10	0.03	Small

S2 Effects of ML Region Size and Embedding on Protein-Ligand Binding

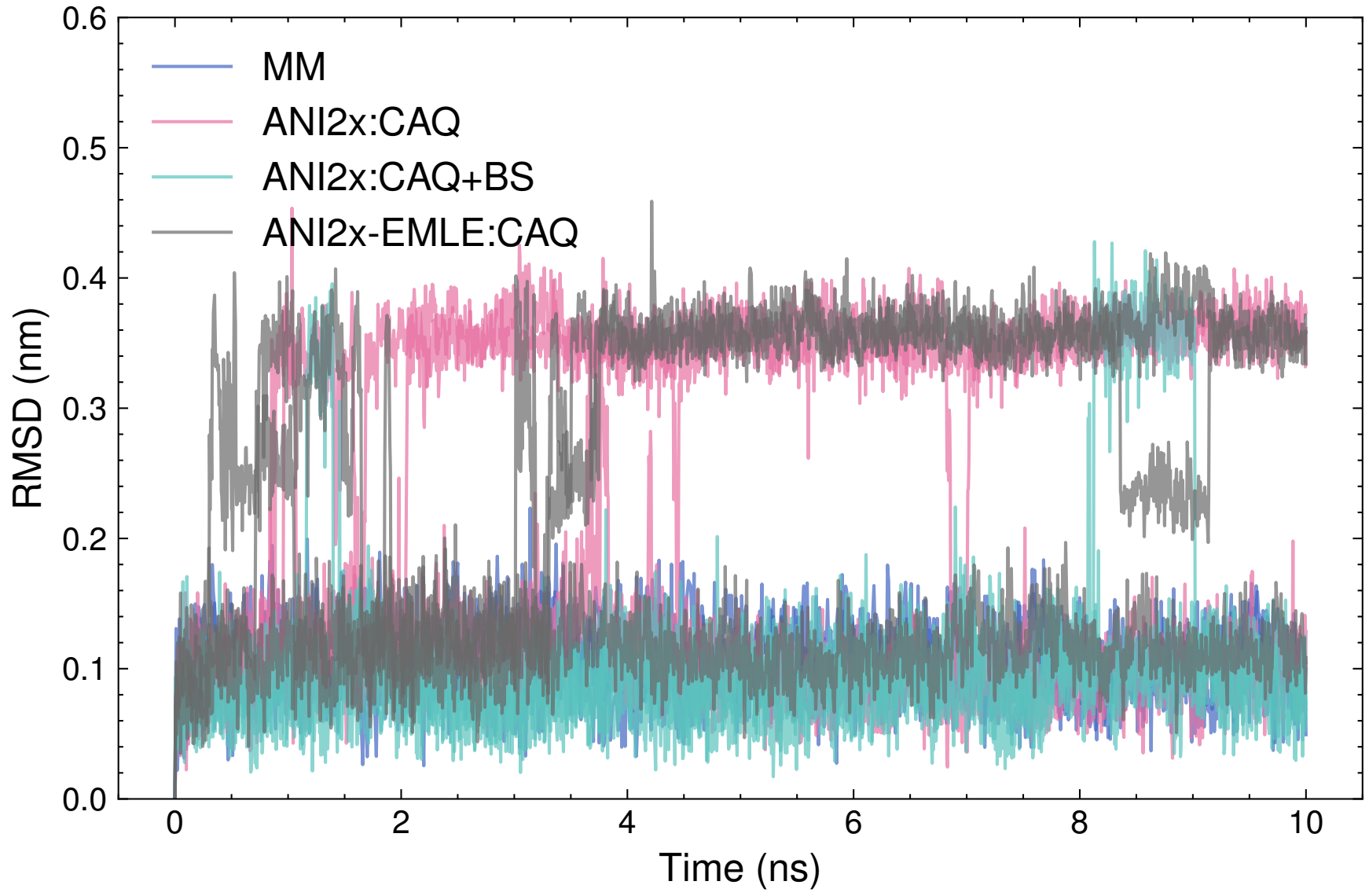

Figure S1: RMSD calculated with respect to the equilibrated starting structure for three replicates of one pure MM and three ML/MM simulations: Using ANI2x for the catechol ligand (CAQ), ligand plus binding site (CAQ+BS), and EMLE for catechol.

Solvation Structure of Phenol in Water

Here, we briefly discuss the solvation structure of phenol in water. With its relatively simple structure and hydrogen bonding OH-site, it is a good candidate to investigate also the effects of an electrostatic embedding model like EMLE,[1] and is readily comparable with previous results.[2] The corresponding RDF is shown in Fig. S2. We here expand on the results in Ref.[2] by extending the simulation time to 200 ps, including a different base NNP model (MACE) as well as an alternative electrostatic embedding (EMLE).

To perform the simulations, we first created a simulation box with a phenol molecule parametrized with the General Amber Force Field (GAFF)[3] and solvated with 3991 TIP3P[4] water molecules. Energy minimization was performed for 10000 steps, after which the system was allowed to equilibrate for 100 ps in the NVT and 100 ps in the NPT ensemble using

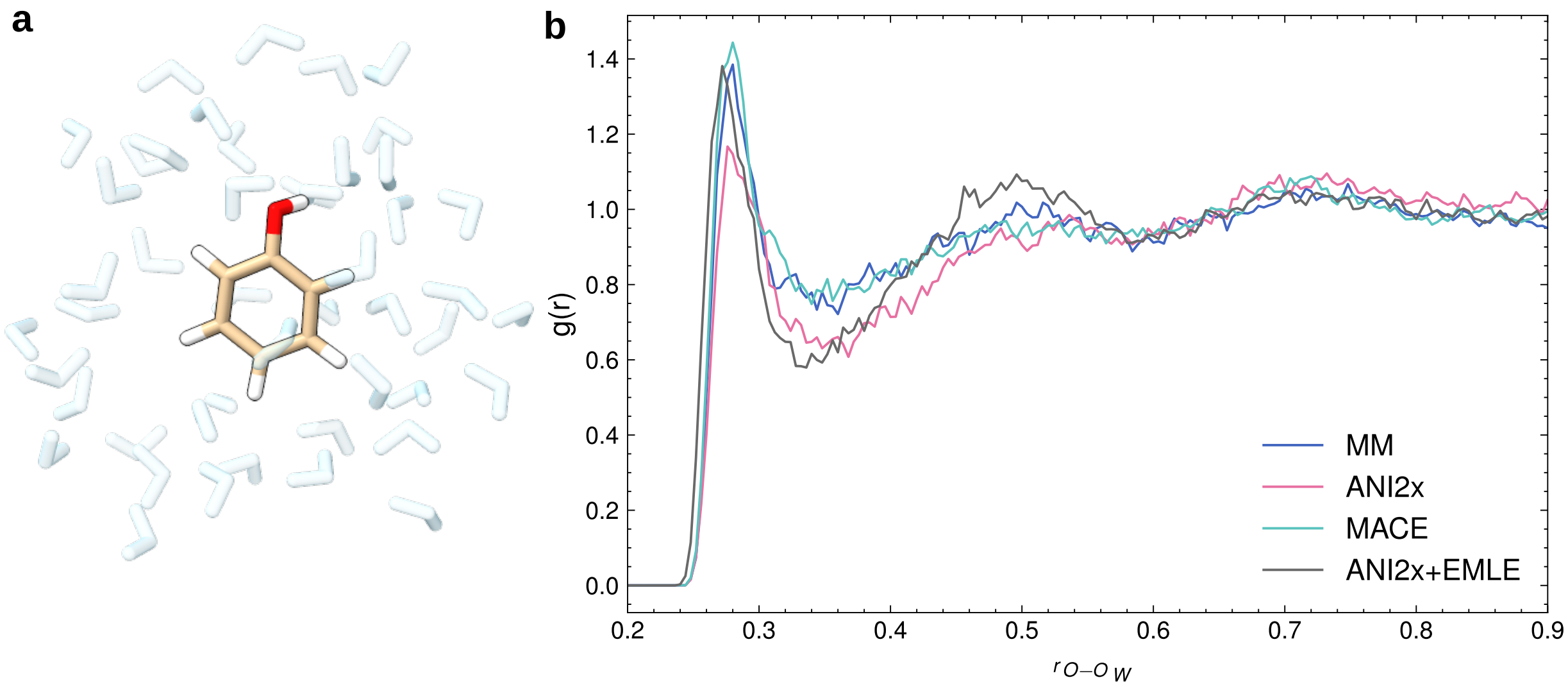

Figure S2: Solvation structure of phenol in water. (a) Structure of phenol molecule with surrounding water molecules. (b) Solvation structure of phenol in water, showing RDF between the phenol oxygen and water oxygens, calculated from 100 ps MD simulations for a pure MM description and ML/MM simulations with ANI2x, MACE and ANI2x-EMLE models, respectively.

stochastic velocity and cell rescaling[5,6] at 300 K and 1 bar, respectively, as well as a time step of 2 fs. Production simulations in the NVT ensemble were carried out from the final conformation of the NPT equilibration for 200 ps at a time step of 0.5 fs for pure MM, ANI-2x/MM, MACE/MM and EMLE/MM descriptions. For the calculation of the RDFs, which was performed with the `gmx rdf` command line tool, the first 5 ps were discarded from further analysis.

It can be seen that treating the phenol molecule with the ANI-2x or MACE potential, as opposed to pure MM, does not seem to have a significant effect on the RDF. This is to be expected, as the interaction with the water molecules surrounding the OH-group is modeled with classical electrostatic and Lennard-Jones potentials in all three of these cases.
In the case of the EMLE model, we do see a significant departure from this behavior, as the first peak is shifted slightly to the left and sharper, a result in line with previous results.[2] Furthermore, we see stronger coordination in the second hydration shell, which is accessible to us because of longer simulation times. In terms of computational performance, the MM

production simulation ran at 200 ns/day, ANI-2x optimized with NNPOps at 29 ns/day, MACE at 2.6 ns/day and EMLE at around 3.6 ns/day, all at a timestep of 0.5 fs. We note however, that a QM/MM simulation of the same system on comparable hardware would exhibit a performance on the order of *ps/day*. In contrast, even with the slowest modules, our results were obtained within a few hours of simulation wall-time.

S3 Performance Scaling of Water Boxes

Table S2: Performance benchmarks for water boxes of increasing size in terms of walltime per step (ms). Results were obtained on a NVIDIA RTX 3070 GPU at 32-bit floating point precision.

Atoms	ANI-2x	MACE-OFF-s	AIMNet2	NNPOps	Nutmeg
3	8.755	11.253	7.130	0.973	9.339
9	8.110	10.925	7.191	1.018	9.112
21	8.625	10.883	7.320	1.611	10.411
30	8.715	10.871	7.495	2.094	10.445
90	9.446	12.034	7.495	5.226	13.867
210	10.281	13.850	7.626	11.621	24.898
300	9.839	16.768	9.635	16.377	33.461
900	10.687	59.991	70.967	–	89.567
2100	12.746	153.515	–	–	198.964
3000	16.019	221.271	–	–	297.982
9000	38.068	–	–	–	840.676
21 000	90.338	–	–	–	–
30 000	133.865	–	–	–	–

Table S3: Performance benchmarks for water boxes of increasing size in terms of throughput (ns/day) at a 1 fs time step. Results were obtained on a NVIDIA RTX 3070 GPU at 32-bit floating point precision.

Atoms	ANI-2x	MACE-OFF-s	AIMNet2	NNPOps	Nutmeg
3	9.869	7.678	12.117	88.840	9.252
9	10.654	7.908	12.014	84.882	9.482
21	10.017	7.939	11.804	53.632	8.299
30	9.914	7.948	11.528	41.254	8.272
90	9.147	7.180	11.528	16.534	6.231
210	8.404	6.238	11.329	7.435	3.470
300	8.781	5.153	8.968	5.276	2.582
900	8.085	1.440	1.217	–	0.965
2100	6.779	0.563	–	–	0.434
3000	5.394	0.390	–	–	0.290
9000	2.270	–	–	–	0.103
21 000	0.956	–	–	–	–
30 000	0.645	–	–	–	–

References

(1) Zinovjev, K. Electrostatic Embedding of Machine Learning Potentials. *Journal of Chemical Theory and Computation* **2023**, *19*, 1888–1897.

(2) Semelak, J. A.; Pickering, I.; Huddleston, K.; Olmos, J.; Grassano, J. S.; Clemente, C. M.; Drusin, S. I.; Marti, M.; Gonzalez Lebrero, M. C.; Roitberg, A. E.; Estrin, D. A. Advancing Multiscale Molecular Modeling with Machine Learning-Derived Electrostatics. *Journal of Chemical Theory and Computation* **2025**,

(3) Wang, J.; Wolf, R. M.; Caldwell, J. W.; Kollman, P. A.; Case, D. A. Development and Testing of a General Amber Force Field. *Journal of Computational Chemistry* **2004**, *25*, 1157–1174.

(4) Jorgensen, W. L.; Chandrasekhar, J.; Madura, J. D.; Impey, R. W.; Klein, M. L. Comparison of Simple Potential Functions for Simulating Liquid Water. *The Journal of Chemical Physics* **1983**, *79*, 926–935.

(5) Bussi, G.; Donadio, D.; Parrinello, M. Canonical Sampling through Velocity Rescaling. *The Journal of Chemical Physics* **2007**, *126*, 014101.

(6) Bernetti, M.; Bussi, G. Pressure Control Using Stochastic Cell Rescaling. *The Journal of Chemical Physics* **2020**, *153*, 114107.
